# Microbiome research for advancing engineering in life science

**DOI:** 10.1002/elsc.202400028

**Published:** 2024-04-05

**Authors:** Feng Ju, Qixiao Zhai, Gang Luo, Hongzhi Tang, Lei Dai

**Affiliations:** ^1^ Environmental Microbiome and Biotechnology Laboratory (EMBLab) School of Engineering Westlake University Hangzhou China; ^2^ Westlake Laboratory of Life Sciences and Biomedicine, School of Life Sciences Westlake University Hangzhou China; ^3^ School of Food Science and Technology Jiangnan University Wuxi Jiangsu China; ^4^ Department of Environmental Science and Engineering Fudan University Shanghai China; ^5^ State Key Laboratory of Microbial Metabolism Joint International Research Laboratory of Metabolic and Developmental Sciences, School of Life Sciences and Biotechnology Shanghai Jiao Tong University Shanghai China; ^6^ CAS Key Laboratory of Quantitative Engineering Biology, Shenzhen Institute of Synthetic Biology Shenzhen Institute of Advanced Technology, Chinese Academy of Sciences Shenzhen China

Microbiome research has become increasingly prominent, as scientists explore the intricately assembled microbial communities (i.e., microbiota) and their wide‐ranging impacts on human systems (e.g., health and foods), environmental sustainability (bioremediation, biogeochemistry, and ecosystem biorestoration, or 3B for Sustainability), and next‐generation bioeconomy (i.e., bioenergy, biomedicine, and biomaterials, or 3B for Resources). This burgeoning field has been driven by the widespread adoption of meta‐omics methodologies, such as metagenomics, metatranscriptomics, metaproteomics, and metabolomics. In this special issue, we present a compendium of recent human and environmental microbiome studies that elucidate the multifaceted roles of microbial communities and their implications across different domains of research in life sciences and related fields of application.

The gut microbiome stands out as a central player in human health, influencing fundamental physiological processes such as digestion, immunity, and metabolism. Tang et al. delves into the intricate interplay between the gut microbiota and the host epigenome in the context of Non‐alcoholic Fatty Liver Disease (NAFLD), shedding light on how microbial factors can modulate gene expression patterns associated with NAFLD pathogenesis [1]. Similarly, Zoghi et al. investigate the association between gut dysbiosis and nutritional imbalances in children, underscoring the potential therapeutic avenues for modulating gut microbiota composition to restore energy homeostasis [2].

**FIGURE 1:**
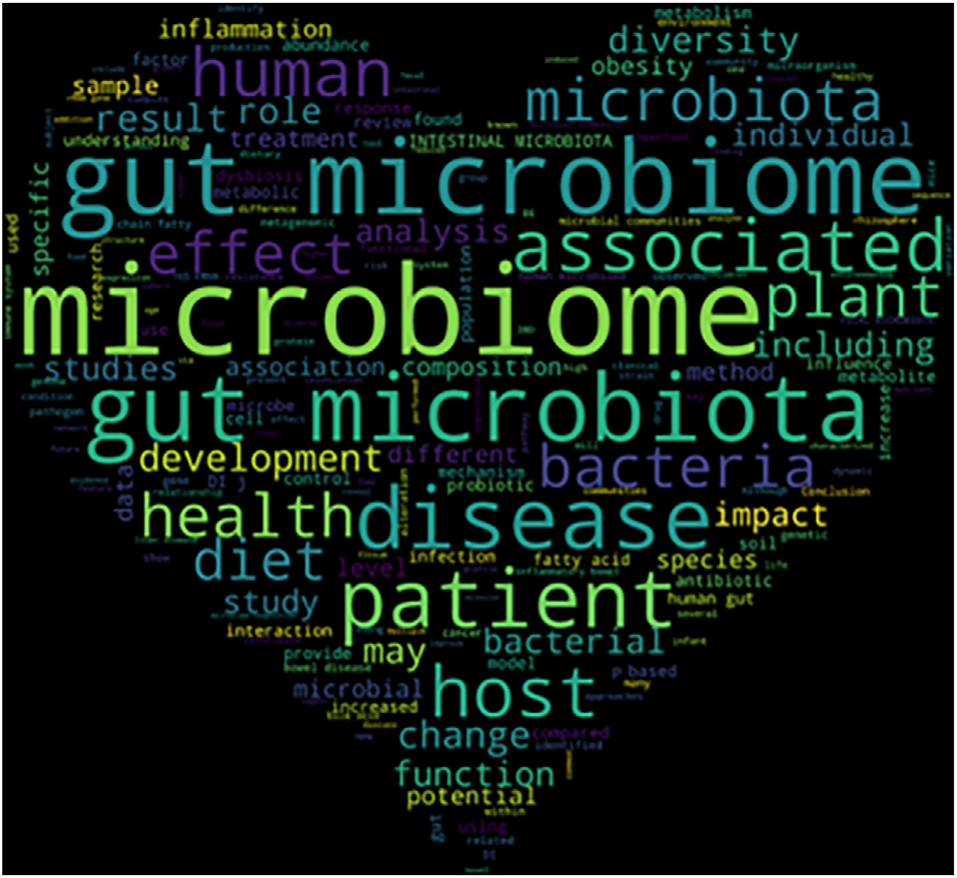
A word cloud for microbiome research based on the title and abstract search of 1300+ highly cited papers in Web of Science as of April 14, 2022.

Moreover, the symbiotic interplay between flavonoids and the gut microbiota emerges as a promising area of study in maintaining metabolic balance and overall health by Zhou et al. [3]. Flavonoids, abundant in fruits and vegetables, serve as essential dietary components that undergo biotransformation by gut microbes, yielding bioactive metabolites with various health‐promoting properties. Understanding this intricate interplay opens new avenues for leveraging dietary interventions to modulate gut microbiota composition and enhance metabolic health (Figure 1).

Beyond human health, microbial communities also play critical roles in environmental processes, particularly in the biodegradation of pollutants. Huang et al. leverage meta‐omics approaches to uncover the genetic potential of microbial communities in contaminated environments, offering insights into potential bioremediation strategies for mitigating environmental pollution [4].

Furthermore, microbiomes offer promising avenues for bioconversion and biodegradation processes in the context of biotechnology and industrial applications. Zhu et al. investigate the dynamics of microbial consortia during primary sludge and food waste fermentation, revealing insights into how different environmental conditions and additives can modulate fermentation product profiles [5]. The study demonstrates the product plasticity of the microbiome fermentation process and suggests a promising solution for future biowaste valorization. Similarly, Wu et al. examine the effects of varying H_2_/CO_2_ ratios on microbial community composition and product distribution, emphasizing the importance of microbial community dynamics in bioprocess optimization [6].

Lastly, Xu et al (2023) showcase the utility of synthetic microbial communities (SynComs) in efficiently managing high‐salt and oily food waste through solid‐state aerobic biodegradation. Their study highlights the potential of engineered microbial consortia for sustainable waste management and resource recovery [7].

In summary, the studies presented in this special issue underscore the intricate interplay between microbial communities and various aspects of human health, environmental sustainability, and industrial processes. As microbiome research continues to advance, it holds immense promise for addressing pressing societal, environmental, and global sustainability challenges, and fostering innovation across interdisciplinary frontiers underlying the next‐generation bioeconomy in the fields of industrial biotechnology, health and medicine, food and agriculture, environmental biotechnology, and bioenergy.
